# Comprehensive Maturity Onset Diabetes of the Young (MODY) Gene Screening in Pregnant Women with Diabetes in India

**DOI:** 10.1371/journal.pone.0168656

**Published:** 2017-01-17

**Authors:** Mahesh Doddabelavangala Mruthyunjaya, Aaron Chapla, Asha Hesarghatta Shyamasunder, Deny Varghese, Manika Varshney, Johan Paul, Mercy Inbakumari, Flory Christina, Ron Thomas Varghese, Kurien Anil Kuruvilla, Thomas V. Paul, Ruby Jose, Annie Regi, Jessie Lionel, L. Jeyaseelan, Jiji Mathew, Nihal Thomas

**Affiliations:** 1 Department of Endocrinology, Diabetes & Metabolism, Christian Medical College, Vellore, India; 2 Department of Neonatology, Christian Medical College, Vellore, India; 3 Department of Obstetrics and Gynaecology, Christian Medical College, Vellore, India; 4 Department of Biostatistics, Christian Medical College, Vellore, India; University of Bonn, Institute of Experimental Hematology and Transfusion Medicine, GERMANY

## Abstract

Pregnant women with diabetes may have underlying beta cell dysfunction due to mutations/rare variants in genes associated with Maturity Onset Diabetes of the Young (MODY). MODY gene screening would reveal those women genetically predisposed and previously unrecognized with a monogenic form of diabetes for further clinical management, family screening and genetic counselling. However, there are minimal data available on MODY gene variants in pregnant women with diabetes from India. In this study, utilizing the Next generation sequencing (NGS) based protocol fifty subjects were screened for variants in a panel of thirteen MODY genes. Of these subjects 18% (9/50) were positive for definite or likely pathogenic or uncertain MODY variants. The majority of these variants was identified in subjects with autosomal dominant family history, of whom five were in women with pre-GDM and four with overt-GDM. The identified variants included one patient with *HNF1A* Ser3Cys, two *PDX1* Glu224Lys, His94Gln, two *NEUROD1* Glu59Gln, Phe318Ser, one *INS* Gly44Arg, one *GCK*, *one ABCC8* Arg620Cys and one *BLK* Val418Met variants. In addition, three of the seven offspring screened were positive for the identified variant. These identified variants were further confirmed by Sanger sequencing. In conclusion, these findings in pregnant women with diabetes, imply that a proportion of GDM patients with autosomal dominant family history may have MODY. Further NGS based comprehensive studies with larger samples are required to confirm these finding

## Introduction

Diabetes mellitus(DM) has evolved into a global epidemic and as Asian countries have undergone an economic, social and nutritional transition, type 2 diabetes mellitus and gestational diabetes mellitus (GDM) have increased exponentially[1]. GDM is defined as “carbohydrate intolerance of any degree of severity with an onset or first recognition during pregnancy”[2]. The prevalence of GDM ranges from 3.8 to 21%, worldwide. In India, it may affect around 200,000 pregnant women annually[2,3]. In a significant proportion of women, glucose intolerance during gestation appears to resolve after delivery. However, up to 50% of these women develop type 2 diabetes within five years after parturition[4]. Women who develop GDM early (<20 weeks) may have an additional underlying deficit, this may fall into one of three major categories: pre-existing insulin resistance, autoimmune induced disorders and monogenic disease like MODY[5]. With an increasing incidence of early onset diabetes and a high prevalence of GDM[3], there could be a significant proportion of MODY which may be misdiagnosed among pregnant women in India. Traditionally, MODY has been thought to account for 2% of diabetes and results from mutations in one of the thirteen genes that have been reported till date[6]. In addition, MODY gene screening would reveal not only those genetically predisposed women, but also their offspring who are at a 50% risk of inheriting the mutation and subsequently may enable the ability to predict the potential response to sulphonylureas (SU) in some patients[7] and aid in identifying the commodities in some forms of MODY[8].

The standard approach for diagnosis of MODY includes sequential screening of the first three common MODY genes which include hepatocyte nuclear factor 1alpha (*HNF1A*), hepatocyte nuclear factor 4 alpha (*HNF4A*) and Glucokinase (*GCK*)[9]. In addition, studies on MODY in pregnant women with diabetes were largely limited to the screening of *GCK*, *HNF1A* and *HNF4A* genes[10]. However, with the advent of next-generation sequencing(NGS) technology, there has been a significant improvement in the speed and scalability of sequencing[11]. Utilizing NGS based strategy, we have recently reported a wider spectrum of MODY mutations involving *NEUROD1* and *PDX1* in patients with young onset diabetes (<30years) in India[12]. Recognizing the presence and pattern of MODY genetic component in pregnant women with diabetes, will help in the identification of susceptible individuals, which in turn, may enable preventive and therapeutic measures for both the mother and the developing foetus[13]. In the present study using a similar NGS based approach[12], we have screened for pathogenic variants in a panel of 13 MODY genes in pregnant women with diabetes.

## Methods

In the present cross-sectional observational study, we have recruited women with pregnancy complicated by hyperglycemia, from September 2012 to December 2013. Written informed consent was obtained from all study participants and the study was approved by the Institutional Review Board (IRB), Christian Medical College, Vellore (IRB Min.No.7998/2012). All pregnant women with any degree of glucose intolerance with an age of onset of disease ≤ 35 years and a body mass index (BMI) ≤ 30kg/m2 were included. Though MODY has been traditionally perceived to present before the age of 25 years, the diagnosis of diabetes may be delayed beyond 35 years[14,15]. Further, many MODY subjects in a previous study at our center had a higher BMI of up to 30kg/m^2^ with phenotypic characteristics that were typical of MODY and had a reduced insulin secretion when compared to subjects with type 2 diabetes and normal controls[12]. Those with an age of onset of diabetes >35 years or a body mass index (BMI) >30kg/m^2^, type 1 diabetes mellitus (T1DM) assigned based on the presence of glutamic acid decarboxylase (GAD)antibody and/or low C-peptide levels (<0.6ng/ml), secondary diabetes or fibrocalcific pancreatic diabetes were excluded from the study. We selected control subjects from a homogenous population of Dravidian ethnic origin (South Indian, Tamil speaking). All the control subjects had a normal OGTT with an HbA1c <5.7. Further, these subjects were without any known family history of diabetes. The body mass index was calculated from either the pre-conceptional weight or the weight recorded at the initial visit within 8 weeks of gestation. The biochemical assays were performed on a Hitachi 912 auto analyzer using reagents from Roche Diagnostics (Mannheim, Germany), GAD antibodies were measured with the radioimmunoassay technique (Bio-Rad iMark™ microplate ELISA reader, intra-assay; CV:5%). The glycosylated haemoglobin (HbA1c) was measured by the ion-exchange base High Performance Liquid Chromatography (HPLC) assay method (Bio-Rad Variant II turbo glycated haemoglobin (HbA1c) analyzer; CV: 2.6%). Based on the International Association of the Diabetes and Pregnancy Study Groups (IADPSG) classification[16] of the hyperglycemic disorders complicating pregnancy, our patients were classified into three groups.

## 1. Gestational Diabetes Mellitus [GDM]

The diagnosis of GDM is made when any of the following plasma glucose values are exceeded after a 75-g oral glucose tolerance test (OGTT) any time during pregnancy,

1. Fasting: ≥92 mg/dl (5.1 mmol/l)
2. 1 h: ≥180 mg/dl (10.0 mmol/l)
3. 2 h: ≥153 mg/dl (8.5 mmol/l)

[NOTE: All women not previously diagnosed with diabetes underwent a 75-g oral glucose tolerance test (OGTT) at 24–28 weeks of gestation. The OGTT was performed in the morning after an overnight fast of at least 8 hours. As per the IADPSG Consensus Panel OGTT was not routinely performed before 24–28 weeks gestation, but a fasting plasma glucose value (lower than those diagnostic of diabetes) in early pregnancy (≥ 5.1mmol/l) was also classified as GDM].

## 2. OvertDabetes Mellitus [Overt-GDM]

Glucose values / HbA1c that were diagnostic of diabetes using the standard American Diabetes Association (ADA) Criteria[16] at the first visit any time after conception or at 24–28 weeks of gestation during the one step 75-g oral glucose tolerance test (OGTT) were categorised as “overt-GDM”. Women who meet the criteria for diabetes are classified as a separate category named“overt diabetes in pregnancy” by the IADPSG criteria and represent the "highest at risk" GDM cohort, who have an increased risk of congenital abnormalities and diabetes related complications[17].

## 3. Pre-Gestational Diabetes Mellitus [PRE-GDM]

Pregnant women who were known to have diabetes prior to conception.

## Statistical analysis

Depending on the selection criteria for screening, the prevalence of MODY in pregnant women with diabetes has been reported to range from 1% to 80%(10). This wide variation in previous studies on the prevalence of MODY mutations may be a result of highly variable inclusion criteria for GDM in order to increase the yield of the test. In this study, we have included a hybrid of patients with and without strong family histories, so a moderate prevalence was hypothesized and hence a 40% "median cumulative prevalence" was assumed and sample size was determined. Therefore, with a median cumulative prevalence of 40% and a 12 to 15% precision, 50 pregnant women were screened with a 13 MODY gene panel (calculated using the N-master software, www.nmaster.cmc-biostatistics.ac.in).

Continuous variables were described using means and standard deviation (SD). For variables with skewed distributions, medians with inter-quartile ranges were used. All categorical variables were summarized by using frequencies and related percentages. The association between pathogenic variants and other covariates (BMI, family history of diabetes) were tested by using Fischer’s exact test, the independent two sample t-test or Chi-square test. The data were analyzed using SPSS version 17.0.

## NGS based MODY gene sequencing

Utilizing our recently published NGS based 2GDMODY protocol(13), we have screened 50 pregnant women with diabetes for a comprehensive panel of 13 MODY genes [*HNF1A*, *HNF4A*, *GCK*, *PDX1*, *HNF1B*, *NEUROD1*, *KLF11*, *CEL*, *PAX4*, *INS*, *BLK*, *ABCC8*,KCNJ11]. Sanger sequencing was performed to confirm all the identified variants of interest. Furthermore, sequencing data from 110 subjects with normal glucose tolerance was utilized for variant interpretation.

## Bioinformatics Analysis

Sequencing was performed on an Ion torrent PGM using the Ion PGM™ 200 Sequencing Kit (Ion Torrent, Life Technologies), utilizing 314 chips (multiplex 4 samples) and 316 chips (multiplex 10–15 samples). The generated sequencing data was mapped to the human genome reference hg19. The coverage analysis was calculated using Torrent Coverage Analysis and potential pathogenic variants were identified using the Torrent Variant Caller and DNA Star software (DNASTAR, Madison, WI, USA). The Human Gene Mutation Database (HGMD^®^Professional 2012.4), was utilized to classify the identified variants as reported or novel. Further, the population sequencing database EXAC [18] was explored to validate the novel variants identified adhering to the latest guidelines by the American College of Medical Genetics19]. The in-silico analysis for likelihood of pathogenicity was performed for all the novel variants using PolyPhen-2[20], Sorting Intolerant From Tolerant (SIFT)[21] and Mutation taster [22] and for sequence conservation with Genomic Evolutionary Rate Profiling (GERP)[23].

## Results

**Fig 1:**
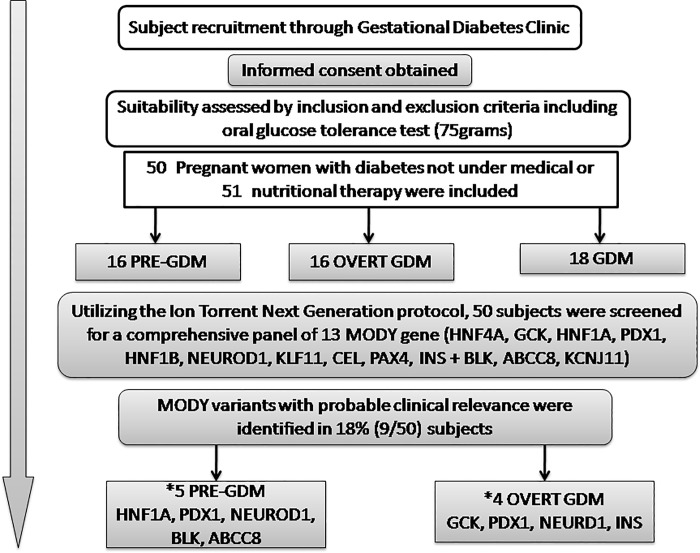
Detailed diagrammatic algorithm of the study with flow chart of NGS screening of the subject.

In the present study 50 subjects were screened, of whom 16 (32%) were with pre-gestational diabetes, 18 (36%) with gestational diabetes and overt diabetes in pregnancy was present in 16 (32%) subjects (Fig 1). There was no consanguinity in this study group and all members were of the Dravidian (south India) ethnicity, the site of recruitment being in the southern most Indian state of Tamil Nadu. The baseline characteristics of these subjects are summarized in Table 1. The mean age of the subjects was 29 years, mean BMI was 27.47kg/m^2^ with 37% (n = 17) below 25kg/m^2^. The median age of diagnosis of diabetes in the pre-GDM group was 22 months prior to conception (except in subject MG101 who was diagnosed at the age of 10 years and was negative for GAD antibodies). The median gestational age of diagnosis in the overt GDM and GDM group was 16 and 20 weeks respectively. The mean HbA1c of the subjects in the pre-GDM group was 8.2% and 7.4% in the overt-GDM group. Overall, 54% (n = 27) of the subjects had a three generation family history of diabetes, who predominantly (n = 20) were in the pre-GDM and overt-GDM groups. Postpartum, patients with overt diabetes in pregnancy (n = 16) who underwent an oral glucose tolerance test and/or HbA1c after 12 weeks post-partum, 25% (n = 4) had diabetes or 31% (n = 5) had impaired glucose tolerance and the rest (44%) were with normal glucose tolerance (n = 7). This was similar to the study by Wong et.al., (17) where about 27% had diabetes and 33% had impaired glucose tolerance with the remaining 40% returning to normal glucose tolerance post-partum. Among those with GDM (n = 18), 88% (n = 16) reverted to normal glucose tolerance with 12% (n = 2) having impaired glucose tolerance.

**Table 1:** Baseline characteristics of the study subjects.

PARAMETERS	TOTAL	GDM	OVERT	PREGDM	p-value
Number of subjects	50	18	16	16	0.102
Age (years)mean ± SD	29.1 ± 5.1	28.2 ± 4.8	27.3 ± 4.8	31.4 ± 5.1	0.905
Age At Diagnosis (years) mean ± SD	28.3 ± 4.7	28.2± 4.8	27.7 ± 5.1	28.5 ± 6.8	NA
Average Week of detection /Duration (Median & range)		20 weeks (8 to 32)	16 Weeks (5 to 32)	#22 months (1 to 60)	
* BMI (kg/m2)	27.47 ± 3.82	26 ± 3.84	29.0 ± 2.56	26.4 ± 4.46	0.218
18.5–23	7 [14%]	2 (11.1%)	1(6.2%)	4(25%)	0.496
23.1–24.9	11[22%]	5(27.8%)	1(6.2%)	1 (6.2%)	
25–29.9	25 [50%]	7(38.9%)	10 (62.5%)	8(50%)	
30–34.9	7[14%]	4 (22.2%)	4(25%)	3(18.7%)	
3 generation family history of diabetes	27 [54%]	7(11.1%)	9(56.2%)	11(68.8%)	0.213
Number of pregnancies					
Primigravida	24 (48%)	11 (61.1%)	7 (43.8%)	6 (37.5%)	
Multigravida (≥ G2)	26 (52%)	7 (38.9%)	9 (56.2%)	10 (62.5%)	
Previous pregnancies					
Previous GDM	5 (20%)	2	1	1	NS
Previous miscarriages	13 (50%)	6	5	2	
Previous congenital anomaly	1 (3.8%)	0	0	1	
Previous LGA	4 (15.4%)	1	1	2	
Previous LSCS	9 (34.6%)	4	4	1	
Glycaemic profile					
Fasting plasma glucose (mg%)[mean± sd]	125 ± 35	98±13	150±24	130 ± 38	<0.0001
2hour post meal Fasting plasma glucose (mg%) (mean± sd)	186 ± 58	147±23	213±51	206 ± 70	<0.003
HbA1c (%) mean± sd	7.18±1.51	5.5±0.5 [n = 13]	7.4±1.2 [n = 13]	8.2±1.3 [n = 15]	<0.0001

*Calculated based on preconception weight / weight recorded at the visit in first trimester.

^#^ All except for one subject who had diabetes for 16 years.LGA / macrosomia = Birth weight of > 90th percentile (≥ 3.50 kg) in the neonates. BMI: Body Mass Index, N/S: not significant, N/A: not applicable. Multigravida (≥G2): a woman that is or has been pregnant for at least a second time. LSCS:

## Next Generation Sequencing based MODY screening

**Fig 2:**
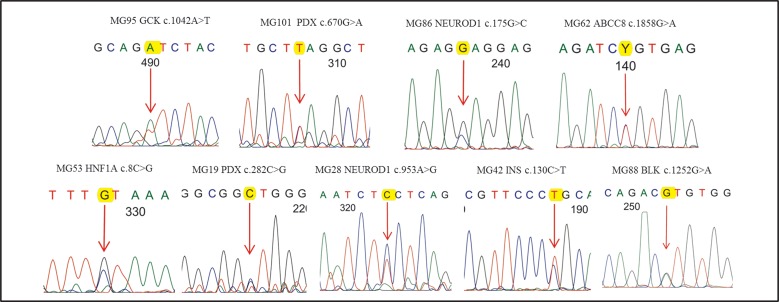
Sanger confirmation of MODY variants identified in the study.

**Fig 3:**
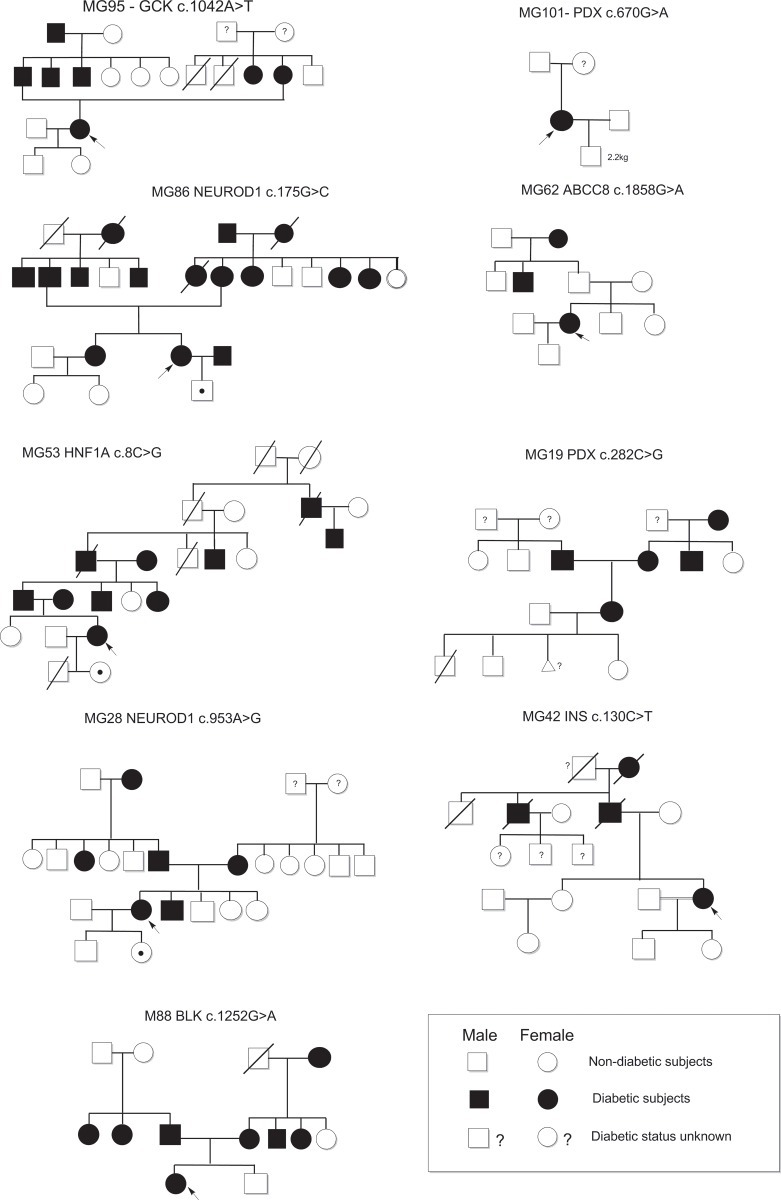
Detailed pedigree charts of MODY positive pregnant women with diabetes.

The mean NGS depth was 536X and >95% of the target was sequenced at a minimum depth of 20X covering all the coding regions. In this study, we found that 18% (9/50) of the pregnant women with diabetes carried MODY related variants. Among these subjects, five were with pre-GDM and four had overt GDM.The mean age at diagnosis of MODY positive subjects was 29.6 years. The mean BMI was 27.1 kg/m^2^ and only two subjects had a BMI of <25kg/m^2^. Overall, there was no significant difference between the subjects (MODY positive and negative) with regards to their age at diagnosis, BMI and birth weight of the offspring. The details of the identified variants along with the Sanger confirmation are summarized in Table 2 & Fig 2 and the pedigree charts in Fig 3. Three of the nine MODY related variants identified in this study were novel and were not present in 110 control subjects and the 1000 genome and the EXAC database. The reported and the novel variants identified have been classified as per ACMG 2015 guidlines based on in-silico analysis[19] and family studies (in five cases).

**Table 2:** Mutations identified by targeted next-generation sequencing in pregnant women with diabetes.

MEPN	AGE	AOD	DOD	GAOD	TYPE	BMI	TP	GENE	Mutation	AA change	FS	SIFT	PP2	MT	COMMENT
MG95	23	23	NA	21 wks	OVERT GDM	24	DIET	GCK	c.1042T>A	Ile348Phe	F	DAM	PD	DC	Likely pathogenic
MG101	26	10	16 yrs	NA	PREGDM	23	INS	PDX	c.670 G>A	Glu224Lys	M	DAM	PD	DC	Pathogenic
MG86	36	36	NA	24 wks	OVERT GDM	30	MET+ SU	NEUROD1	c.175 G>C	Glu59Gln	M, Sis, S	Tol	B	DC	
															Uncertain significance
MG 62	30	24	6 yrs	NA	PREGDM		MET	ABCC8	c.1858G>A	Arg620Cys		DAM	Tol	DC	
															Uncertain significance
MG53	25	22	3 yrs	NA	PREGDM	30	INS	HNF1A	c.8C>G	Ser3Cys*	F, D	DAM	PD	DC	Likely pathogenic
MG19	35	35	NA	12 wks	OVERT GDM	27	MET	PDX	c.282C>G	His94Gln*		Tol	B	DC	Uncertain significance
MG28	29	27	2 yrs	NA	PREGDM	30	MET+ SU	NEUROD1	c.953A>G	Phe318Ser*	F, D	DAM	B	DC	Likely pathogenic
MG42	35	35	NA	28 wks	OVERT GDM	27	DIET	INS	c.130C>T	Gly44Arg		DAM	PD	DC	
															Uncertain significance
MG 88	23	23	4 wks	NA	PRE GDM	30	MET+ INS	BLK	c.1252G>A	Val418Met		DAM	PD	DC	Likely pathogenic

MEPN–Molecular Endocrinology Pin Number, AOD: Age of diagnosis, DOD: Duration of diabetes, GAOD: Gestational age of Diagnosis, BMI: Body mass index, AA: Amino acid, FS: Family members screened, F:Father, M:Mother, Sis: Sister, S: Son, D: Daughter, *Novel variant, SIFT: Sorting intolerant from tolerant; PolyPhen 2:Polymorphism Phenotyping v2; MT: Mutation taster, DAM: Damaging, Tol: Tolerated, PD: Probably damaging, B: Benign, DC: Disease causing, HNF4A:Hepatocyte Nuclear factor 4 alpha; GCK: Glucokinase; HNF1A:Hepatocyte nuclear factor 1 alpha; PDX1: Pancreatic and duodenal homeobox 1; HNF1B:Hepatocyte nuclear factor 1 beta; NEUROD1, Neurogenic differentiation factor 1; INS, Insulin gene, MET: Metformin, SU: Sulfonylurea.

Interestingly, in this study, we have identified mutations/rare variants only in patients with pre GDM and overt GDM. In addition, eight (29.6%) of the 27 subjects with an autosomal dominant family history, were positive for MODY related variants, whereas only one (4.3%) of the 23 subjects without a 2/3 generation family history of diabetes carried a MODY variant. Even in our previous study on MODY revealed likely pathogenic MODY variants only in clinically suspected MODY patients and not in young diabetes subjects who do fit into clinical criteria of MODY. Therefore, we believe that few of these variants described in this study even with uncertain significance (variable penetrance or weaker effect) require further evaluation and functional studies to dissect the role of these variants in diabetes.

## Genotype Phenotype Correlation

In the present study, a patient with pre-GDM MG53 was positive for a novel p.S3C *HNF1A* variant. This variant with a paternal inheritance was further transmitted to her child. Her father, who was diagnosed with diabetes at the age of 32 years is currently managed with SU therapy. MG53 during her first pregnancy, had uncontrolled hyperglycemia and delivered a male neonate with transposition of great vessels (TGV) with pulmonary atresia, who expired at the age of 1 month.

We identified MODY4 *PDX1* variants in two subjects, MG19 with overt diabetes and MG101 with pre-gestational diabetes. M19 was positive for a novel H94Q *PDX1* variant and following delivery, requires SU therapy for glycemic control. Her previous pregnancies were complicated by miscarriage and a low birth weight neonate with an early postnatal death. MG101 was positive for a reported E224K *PDX1* pathogenic variant [24] and was diagnosed with diabetes at the age of 10 years with ketoacidosis at onset, negative for autoantibodies and has been on Insulin therapy. She delivered a preterm baby with low birth weight (2240 grams) who was negative for the *PDX1* pathogenic variant. Interestingly, her mother who is a carrier of this *PDX1* E224K has impaired glucose tolerance.

MG86 with overt GDM and MG28 with pre-GDM were found to positive for a reported E59Q variant and a novel F318S *NEUROD1* (MODY-6) variant respectively. MG86 with a BMI of 30kg/m^2^ was diagnosed to have diabetes at 12 weeks postpartum and is currently on metformin with glipizide. Her child is also a carrier of this variant. She has inherited this variant from her mother who was diagnosed to have diabetes at the age of 40 years and had delivered a macrosomic baby with a birth weight of 4000 grams in 1974 at the age of 21 years. MG28, with a novel F318S *NEUROD1* likely pathogenic variant is obese with a BMI of 30kg/m^2^and has a paternal inheritance and her first offspring is a carrier with low birth weight.

MG42 with overt GDM was positive for a novel G44R *INS* gene variant. Postpartum she has impaired glucose tolerance and has been managed with dietary modification alone. Both her parents, her grandfather and paternal uncle who had diabetes, have expired. Her children are negative for this variant.

MG88 had pre GDM and was identified to have a novel *BLK* likely pathogenic variant. Postpartum she had diabetes and is managed with metformin. She is obese with a BMI of 30kg/m^2,^ and similar reports of patients with *BLK* mutation and obesity have been published by Kim et.al [25]

MG95 was positive for a reported *GCK* Ile348Phe mutation and was diagnosed with overt diabetes when screened at 21 weeks of gestation with a HbA1c of 7%. She had GDM in her previous pregnancy and was managed with Insulin. She presented with recurrent gestational diabetes with postpartum normal blood glucose. She had inherited this variant from her father who was diagnosed at the age of 40 years and is currently being managed with SU therapy. The clinical phenotype in carriers of the GCK variant in the present family has been heterogeneous without the characteristic fasting hyperglycemia as seen in other patients with GCK gene mutation[26].

MG62, a 30 year old woman with pre-GDM was positive for Arg620Cys *ABCC8* variant[27] which was previously reported in congenital hyperinsulinism. She was diagnosed with diabetes six years prior to conception and has been managed with metformin and SU therapy. This subject with an ABCC8 variant has early onset of diabetes without a family history of diabetes or hypoglycemia.

## Discussion

Utilizing the NGS based 2GDMODY protocol[12] we have screened a comprehensive panel of thirteen MODY genes in 50 pregnant women with diabetes and identified MODY related variants in 9(18%) subjects. These variants were detected in subjects with overt GDM and pre-GDM (9/32) and the women with GDM were negative for novel or reported pathogenic variants (0/18). The word “overt GDM” has been used for the diagnosis of “overt diabetes in pregnancy” based on the guidelines released by the Association of Diabetes and Pregnancy Study Groups (IADPSG) consensus panel in 2010[16] and The Endocrine Society guidelines on diabetes and pregnancy in 2013[28]. The reason for earlier detection of the disorder during pregnancy at our centre is a result of robust screening done at the first visit since the IADPSG guidelines are followed. These women would have been labelled as GDM if screening had been carried out based on the previous criteria at 24–28 weeks[16]. As previously defined, GDM also included a subgroup with more severe hyperglycemia that presented with special issues concerning management during pregnancy and postpartum follow-up.

Women who meet the criteria for diabetes are now classified as a separate category named “overt diabetes in pregnancy” by the IADPSG criteria and represent the "highest at risk" GDM cohort, who have an increased risk of congenital abnormalities and diabetes related complications. The future risk of these women for developing diabetes appears unclear at present[29]. A retrospective study of 254 women meeting the criteria for overt diabetes has demonstrated that 41% had a normal glucose tolerance at 6–8 weeks postpartum and hence all women classified as “overt diabetes in pregnancy” cannot be considered as having pre-existing diabetes prior to conception[17]. It would indeed be useful to look at this group carefully in future studies on hyperglycemic disorders in pregnancy. A further prospective follow-up comparing women meeting both sets of the IADPSG criteria would therefore be useful in further refining the risk in these patients.

In GDM patients, MODY related variants were present seven times higher (29.6% compared to 4.3%, OR = 9.2, 95% CI 1.06–80.93, P = 0.027) in those with a three generation family history of diabetes. This is similar to earlier reports, wherein the selective screening for pathogenic variants in subjects with the typical phenotype with the presence of a family history of diabetes has shown a prevalence which ranges from 12–80% [10]. Recent study by Flannick et.al., showed that among the general population only 32% of of the subject’s positive for HGMD MODY variants and 31% with likely pathogenic variants had IFG or diabetes[30]. However, when these individuals preselected based on the phenotype, carried an excess low frequency nonsynonymous variants (4.7% compared to 1.5%, OR = 3.2, *P* = 0.011) and an apparent excess of likely pathogenic or HGMD MODY variants in obese old subjects with diabetes when compared to young lean diabetes subjects[30]. These results suggest that the classification of the identified MODY variants needs to based on the phenotype and require further family screening before filtering these variants based on Exac data sets which does contain individual with disease.

*NEUROD1* variants (MODY 6) have been reported from only 10 families [12,31–35]. However, in contrast to this study, the Norwegian study [31] did not find *NEUROD1* as a candidate gene in GDM. Although the clinical phenotype of some *PDX1* and *NEUROD1* variants remains unclear, increased body weight has been reported in earlier studies in subjects with *NEUROD1* mutation [32–34]. Indeed, the mean BMI of the subjects who were *NEUROD1* positive (30kg/m2) in our study was higher than those with other MODY related variants (26.1kg/m2), which signifies the additional role of diabetogenic factors in MODY. Twelve weeks postpartum, in these subjects with *NEUROD1* variants and obesity, we performed an oral glucose tolerance test; both showed a significant reduction in insulin release at 1 and 2 hours when compared with T2D and control subjects (S1 Fig).

MG101 with pre-GDM was positive for a reported E224K *PDX1* mutation which was shown to result in reduced tranactivation and was also found to co-segregating with early-onset diabetes or impaired glucose tolerance in an Indo-Trinidadian family[24]. Further, the E224K, was shown to disrupt the ability of nuclear factor *PCIF1* (PDX C-terminus Interacting Factor-1) to inhibit *PDX1* transactivation, suggesting that the interaction between *PDX1* and *PCIF1*are required for normal glucose homeostasis[36]. However, with 190 allele carreiers in 1000 genome and Exac database[18] could mislead the interpretation of this variant as benign. But with the evidence from functional studies[24] and with recent findings of digenic mutations *NEUROD1* –PDX1[12] included in annotated digenic diseases database (DIDA) identifiers: dd220[37,38] suggest that this variant affects the beta cell functioning and may requires additional genetic or environmental factors to express the disease phenotype.

Subject MG19 with overt-GDM who was positive for a novel H94Q *PDX1* variant had persistently elevated glucose levels in the diabetic range post-partum and is currently being managed with metformin and SU therapy. Further, PDX1 variant carriers MG101 & MG19 had delivered babies with low birth weight. Gragnoli C et.al., in their study found that four out of five pregnant women who were carriers of the *PDX1* pathogenic variant(P33T)[39], had their pregnancies complicated by reduced birth weights, miscarriages, or early postnatal deaths, similar to that noticed in our subjects MG19 and MG101. These findings suggest that the *PDX1* pathogenic variants may provide an increased susceptibility to the occurrence of low birth weight in the offspring of pregnant women with MODY 4 [39]. Therefore, it is important to note the PDX1 variants in pregnant women with diabetes which may predispose these subjects and play a role during gestation and clinical outcomes.

Insulin gene (*INS*) mutations (MODY 10) cause permanent neonatal diabetes (PNDM) and are a very occasional cause of diabetes diagnosed in childhood or adulthood. In the present study, MG42 was positive for a novel G44R *INS* gene variant. She was diagnosed to have overt diabetes in her second pregnancy. In a study by Boesgaard et.al., a novel heterozygous insulin gene variant c.17G>A, R6H was identified in a 27 year old woman with GDM and up to a 30% reduction in beta-cell function measured by an insulinogenic index was noted in the family members(37). The family members with diabetes in this study [40] with insulin gene variants c.17G>A, R6H were not insulin-dependent, as was observed in MG42 in this study.

MG88, a 23 year old woman with a novel *BLK* variant (MODY-11) was obese with a BMI of 30kg/m^2^ similar to earlier reports by Kim et.al., who had noted that there was a higher prevalence of obesity in individuals with diabetes linked to the BLK gene than in diabetic individuals with MODY linked to other loci[25]. Borowiecet et.al., also suggested that the diabetogenic environment conferred by an increased body weight might be necessary for translation of the beta-cell abnormalities caused by the BLK gene variant into diabetes[41].

Further, we have performed Sanger sequencing for all the available first degree relatives of six of the nine women who were positive for MODY related variants and who provided consent for family screening. All the parents and siblings who screened negative for the identified pathogenic variants did not have diabetes. All parents who were positive for the identified variants had diabetes, except the mother of MG101 with a E224K *PDX1* pathogenic variant had impaired glucose tolerance. Further, three of the seven (45%) neonates who were screened were found to carry the identified variant (*GCK*,*HNF1A*,*NEUROD1*). However, the offspring would be labelled as carriers at present and would need long-term follow up. Postpartum, three of the four women with pre-GDM responded to sulphonylureas (SU) whereas one continued on insulin(PDX1); additionally, of the five MODY subjects with overt diabetes in pregnancy who underwent a follow-up oral glucose tolerance test, two of these subjects with *PDX1*, *NEUROD1* progressed to develop diabetes, two subjects with *INS*, *PDX1* showed impaired glucose tolerance, one with a GCK gene variant at 12 weeks postpartum reverted to normal glucose tolerance.

Contemporary literature has reported on MODY pathogenic variants in GDM, suggesting that GCK pathogenic variants are the commonest, followed by *HNF1A*, *HNF4A and PDX1* genes[10]. To date, there were no reports on *NEUROD1*, *INS*, *BLK* and *ABCC8* variants in pregnant women with diabetes. This may be attributable either to the lower prevalence of pathogenic variants in the screened population or due to the sequential screening by the conventional Sanger sequencing methodology, which is limited by cost and scalability. Interestingly, in this study population, utilizing the NGS we have identified MODY variants in *NEUROD1*, *PDX1* and *BLK* genes in comparison to the more commonly reported *HNF4A*, *GCK* and *HNF1A* gene variants. The variable pattern of identified variants in our population in comparison to earlier reports may be attributed to the differential prevalence of various MODY pathogenic variants across the world [12,42]. To the best of our knowledge, this is the first NGS based comprehensive screening of 13 MODY genes in pregnant women with diabetes with the first report of *NEUROD1*, *INS*, *BLK* and *ABCC8* gene variants in pregnant women with diabetes from India.

Phenotypic heterogeneity in MODY is a well-known fact in individual members within a family who share the pathogenic variants has been documented in recent studies[12,26,43]. Furthermore, some of the variants like heterozygous E1506K pathogenic variant in the *ABCC8* gene resulting in congenital hyperinsulinism in infancy, loss of insulin secretory capacity in early adulthood, and autosomal dominant diabetes in middle age in different members[44]. Therefore, parallel NGS approach will help in identifying additional variants in other genes which may contribute to the heterogeneity seen in MODY patients. The novel variants identified in this study were absent in 110 control subjects and the 1000 genome, EXAC database and were predicted to be causative by at least one of the bioinformatics tool. However the absence of these variants in a large number of exomes and variation in the conserved region do not confer pathogenicity, but suggest the need to investigate further to identify the role of these novel variants in diabetes. Indeed, in the absence of detailed familial co-segregation and functional studies, we acknowledge the difficulties and limitations in interpreting the sequencing data in categorizing the novel MODY variants. Further, with thousands of exome sequencing data from the Exac database which includes rare variants causing late onset disorders[18] poses a challenge to classify these variants in conditions such as GDM[45]. However, interpretation of data from NGS based parallel multigene testing would become clinically significant when the genomics data from patients is linked with detailed phenotype and this publication would help in such an effort in MODY and GDM.

## Conclusions

Comprehensive NGS based parallelized multi-gene screening revealed MODY mutations or rare variants only in patients with pre-GDM and overt GDM with an autosomal dominant family history. Therefore, this subset of patients could benefit through MODY gene screening, confirmed diagnosis and appropriate therapy in few cases.

Utilizing the ExAC database resource in variant filtering and classification in young onset or reproductive age onset diseases such as GDM requires caution and need to based on the functional studies, phenotype, and family screening, to confirm the role of these variants in MODY.

We also believe that further family studies and long-term follow up of the offspring would provide an opportunity to understand the heterogeneity, penetrance and pathogenicity of MODY related variants and their response to sulphonylurea treatment. This may also help in deciphering potential implications for treatment during pregnancy and the role of specific pathogenic variants in the occurrence of comorbidities like macrosomia, preterm deliveries, low birth weight, pregnancy loss and neonatal complications. Further, this would enable early detection of glucose intolerance, intervention and appropriate therapy for patients and their families.

## Supporting Information

S1 Fig Insulin secretion in subjects with NEUROD1 mutation. (DOCX)
