# Supplementary material for: Comprehensive Maturity Onset Diabetes of the Young (MODY) Gene Screening in Pregnant Women with Diabetes in India
Source: PLoS One. 2017 Jan 17;12(1):e0168656. doi: 10.1371/journal.pone.0168656 (PMC5240948; doi:10.1371/journal.pone.0168656)

S1 Fig: Insulin secretion in subjects with NEUROD1 mutation**.**


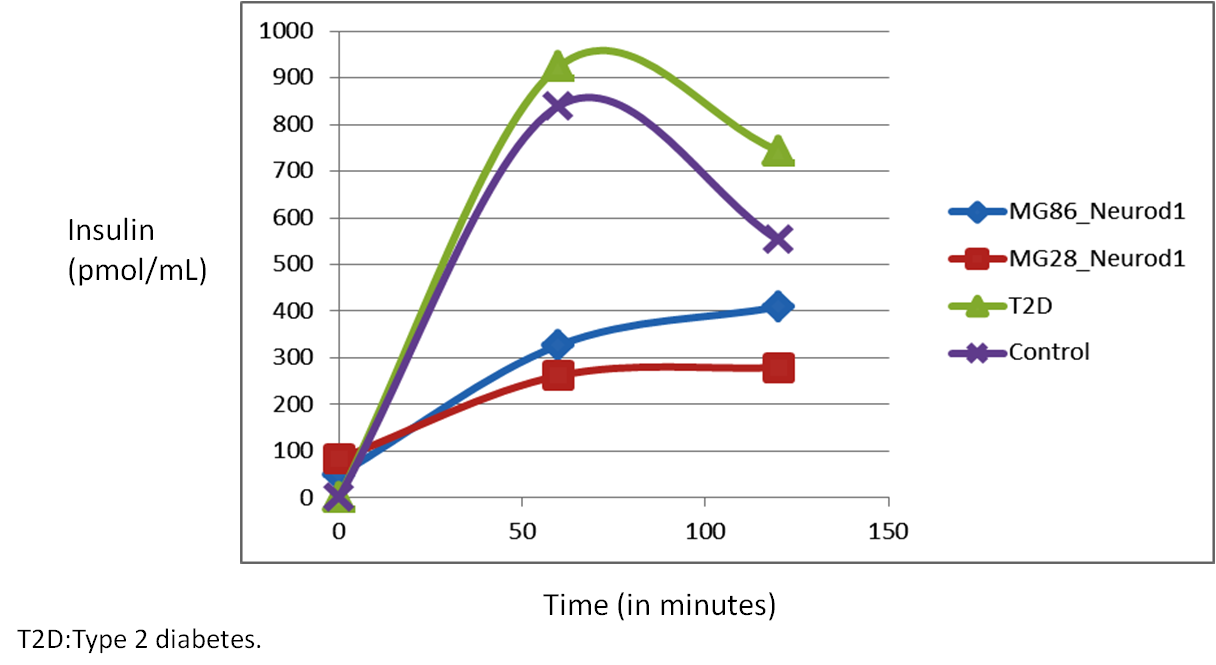


On OGTT, subjects MG28 and MG86 showed a significant reduction in insulin release at 60 and 120 min when compared with controls and Type 2 diabetes (T2D) subjects.

Supplementary figure 2: Suggested algorithm to screen for mutations in
MODY genes in pregnant women with diabetes


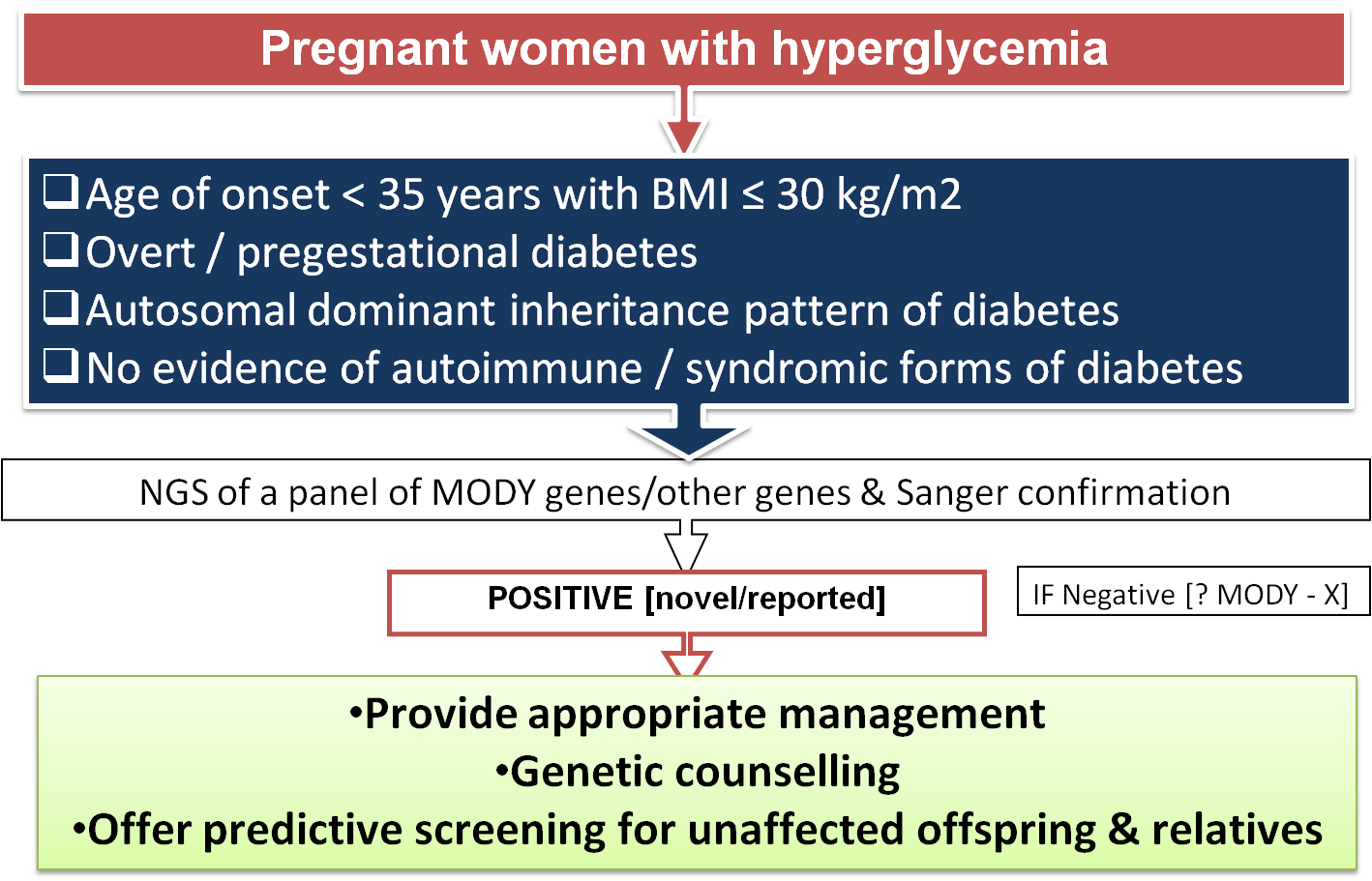

Supplement: S1 Fig — (DOCX) [file pone.0168656.s001.docx]
